# Dietary and Parenteral Nutritional Management of Short Bowel Syndrome with Intestinal Failure in Adults: A Narrative Review

**DOI:** 10.3390/nu18193263

**Published:** 2026-10-02

**Authors:** Brigida Barberio, Beatrice Varotto, Matteo D’Angelo, Lara Tessari, Gianni Di Lecce, Pietro Pirro, Francesco Cesaroni, Sara Cusano, Luisa Bertin, Fabiana Zingone, Patrizia Burra, Luca Busetto, Edoardo Vincenzo Savarino, Giulia Tognana

**Affiliations:** 1 Gastroenterology Unit, Azienda Ospedale-Università of Padova, Department of Surgery, Oncology and Gastroenterology, University of Padova, 35128 Padova, Italy; gianni.dilecce@studenti.unipd.it (G.D.L.); pietro.pirro@studenti.unipd.it (P.P.); francesco.cesaroni@studenti.unipd.it (F.C.); sara.cusano@studenti.unipd.it (S.C.); luisa.bertin.1@studenti.unipd.it (L.B.); fabiana.zingone@unipd.it (F.Z.); burra@unipd.it (P.B.); 2 Clinical Nutrition Unit, Azienda Ospedale-Università of Padova, DIDAS Medicina dei Sistemi, 35128 Padova, Italy; beatrice.varotto@aopd.veneto.it (B.V.); matteo.dangelo@aopd.veneto.it (M.D.); lara.tessari@studenti.unipd.it (L.T.); luca.busetto@unipd.it (L.B.); giulia.tognana@aopd.veneto.it (G.T.); 3 Department of Medicine, University of Padova, 35128 Padova, Italy

**Keywords:** short bowel syndrome, intestinal failure, parenteral nutrition, home parenteral nutrition, intestinal adaptation, oral rehydration solution, GLP-2 analogues, teduglutide

## Abstract

Short bowel syndrome (SBS) is the leading cause of chronic intestinal failure in adults, arising when the residual small bowel can no longer absorb the macronutrients, water, and electrolytes needed to sustain health without intravenous supplementation. Its management is, at its core, a nutritional problem. This narrative review summarises and critically appraises current evidence and guideline recommendations on the two pillars of that management: dietary (oral/enteral) therapy and parenteral nutritional support. We frame both around the two variables that dominate clinical behaviour, namely residual intestinal anatomy and the phase of intestinal adaptation, and translate them into anatomy-tailored dietary strategies, rational fluid and electrolyte replacement, micronutrient surveillance, and the composition, delivery, and complications of (home) parenteral nutrition. We also position the pharmacological adjuncts, culminating in glucagon-like peptide-2 (GLP-2) analogues, within a stepwise strategy to reduce parenteral support dependence. The goal throughout is a practical, physiology-anchored approach that maximises enteral autonomy and quality of life while preventing the metabolic, hepatic and catheter-related complications of long-term intravenous feeding. The literature was identified through structured but non-systematic searches of PubMed/MEDLINE, Embase and the Cochrane Library, and the nature of the evidence supporting each recommendation, ranging from randomised trials to expert consensus, is indicated throughout the text.

## 1. Introduction

Intestinal failure (IF) is defined by ESPEN as the reduction of gut function below the minimum necessary for the absorption of macronutrients and/or water and electrolytes, such that intravenous supplementation is required to maintain health and/or growth [1]. It is graded by onset and expected duration into three functional types: type I, an acute, short-lived, and usually self-limiting condition seen perioperatively or in critical illness; type II, a prolonged acute state in metabolically unstable patients requiring multidisciplinary care over weeks to months; and type III, chronic intestinal failure (CIF) in a metabolically stable patient managed, where possible, at home [1,2].

Short bowel syndrome (SBS), defined in adults as a residual small bowel length in continuity below approximately 200 cm following extensive resection, is the most common pathophysiological mechanism of CIF, accounting for roughly two-thirds of adult cases [3,4]. The dominant causes are Crohn’s disease, mesenteric ischaemia, radiation enteritis, surgical complications and volvulus [5]. Whatever the trigger, the resulting clinical picture is dominated by malabsorption: diarrhoea or high stomal output, steatorrhoea, dehydration, electrolyte and micronutrient depletion, and weight loss [6].

Because the primary deficit is impaired absorption, the management of SBS is fundamentally a nutritional discipline. It rests on two complementary pillars: optimisation of what the patient eats and drinks (dietary/enteral therapy) and replacement of what the gut cannot absorb (parenteral support, PS). This review addresses both, is organised around the two clinical variables that govern almost every decision (residual anatomy and the phase of adaptation) and integrates the pharmacological therapies, now including GLP-2 analogues, that can shift the balance from intravenous dependence toward enteral autonomy [3,4,6].

## 2. Literature Search and Review Methodology

This article is a narrative review and does not follow the methodology of a systematic or scoping review. The literature was identified through searches of PubMed/MEDLINE, Embase and the Cochrane Library from inception to July 2026, using combinations of the terms “short bowel syndrome”, “intestinal failure”, “chronic intestinal failure”, “parenteral nutrition”, “home parenteral nutrition”, “oral rehydration solution”, “intestinal adaptation”, “diet”, “micronutrients”, “lipid emulsion”, “intestinal failure-associated liver disease”, “teduglutide”, “glepaglutide”, “apraglutide” and “GLP-2 analogue”. Reference lists of retrieved articles and current guidelines were searched by hand for additional relevant publications. We considered articles published in English concerning adult patients, giving priority to international guidelines, randomised controlled trials, meta-analyses, prospective cohort studies and large registry series; case reports and small uncontrolled series were considered only where no higher-level evidence was available. Final selection reflected the authors’ judgement of relevance to the clinical questions addressed, in keeping with the narrative format, and the general quality criteria proposed for narrative reviews were followed [7].

**Figure 1:**
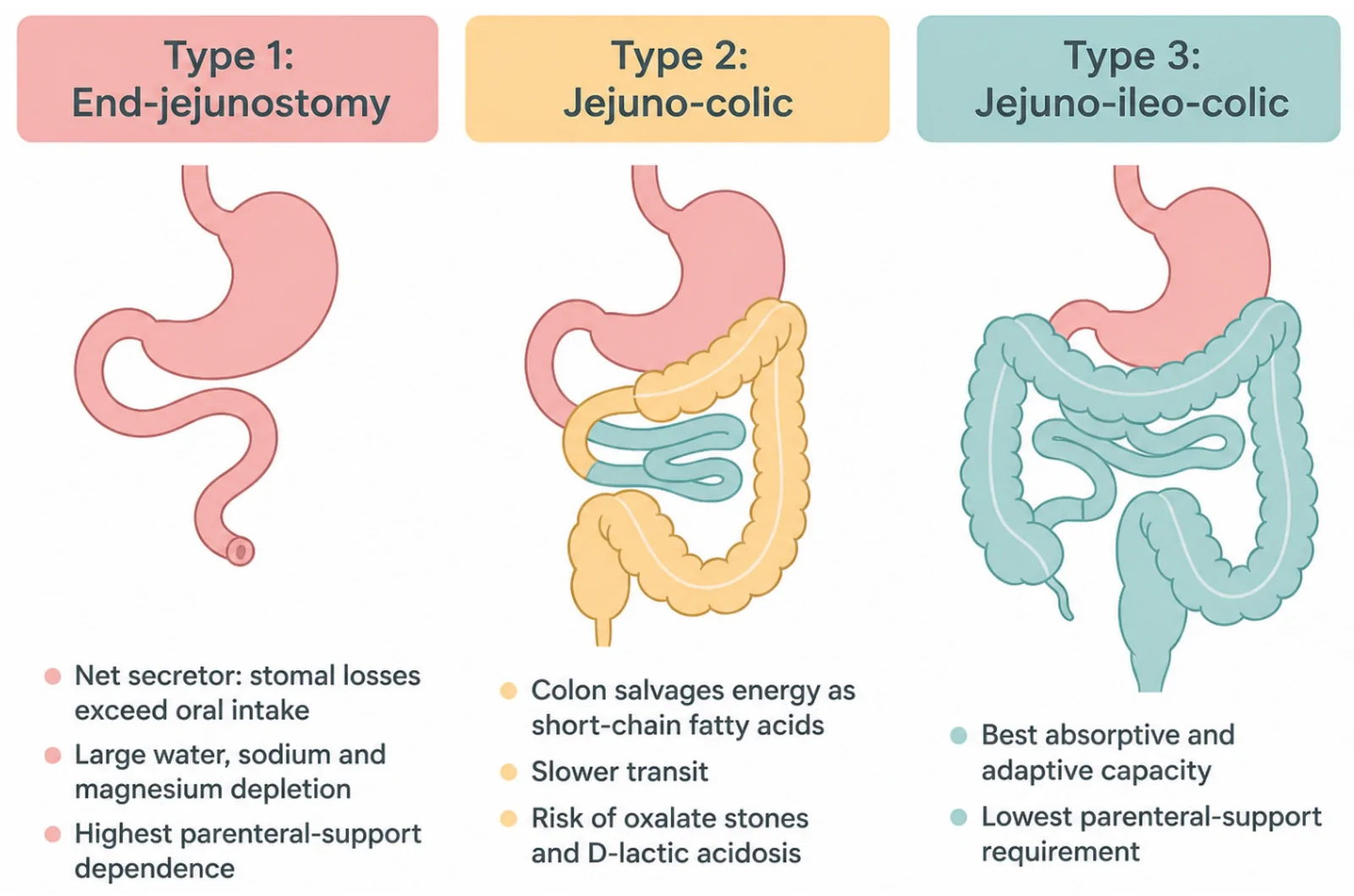
**The three residual anatomies of the short bowel and their functional signature.** The functional consequences (net secretion and high stomal losses in the end-jejunostomy; colonic energy salvage but oxalate stone and D-lactic acidosis risk in colon-in-continuity; preserved adaptation in the jejuno-ileal type) dictate diet, fluid strategy, and parenteral support needs. Original figure designed by the authors for this review; no part of it is reproduced or adapted from published guidelines or illustrations. The graphic was generated with the assistance of a generative artificial intelligence tool (Gemini 2.5 Pro, Google) from content and layout defined by the authors, who checked and edited the output and take full responsibility for its scientific accuracy.

**Figure 2:**
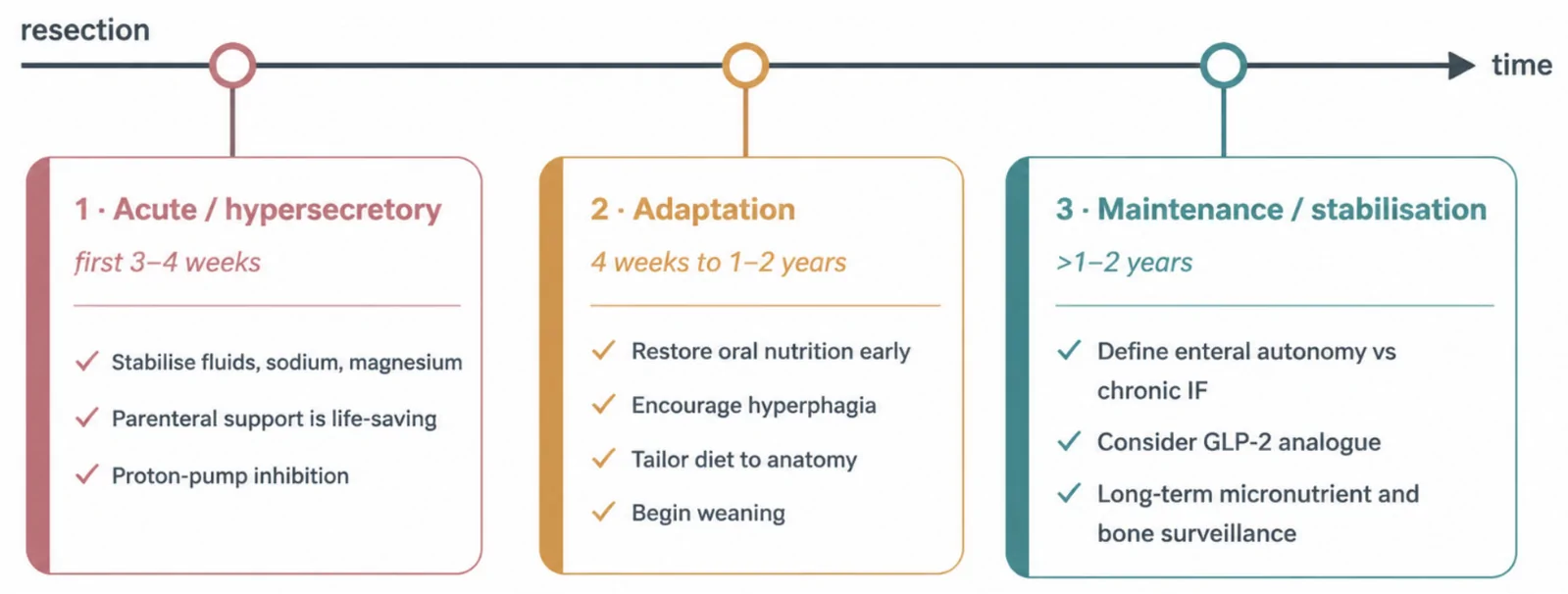
**Phases of intestinal adaptation and the shifting priorities of nutritional care.** Management is not static: fluid and electrolyte stabilisation dominate early, anatomy-tailored diet and weaning dominate the adaptation window, and definition of enteral autonomy versus chronic intestinal failure, with consideration of GLP-2 therapy, dominates the maintenance phase. Original figure designed by the authors for this review; no part of it is reproduced or adapted from published guidelines or illustrations. The graphic was generated with the assistance of a generative artificial intelligence tool (Gemini 2.5 Pro, Google) from content and layout defined by the authors, who checked and edited the output and take full responsibility for its scientific accuracy.

**Figure 3:**
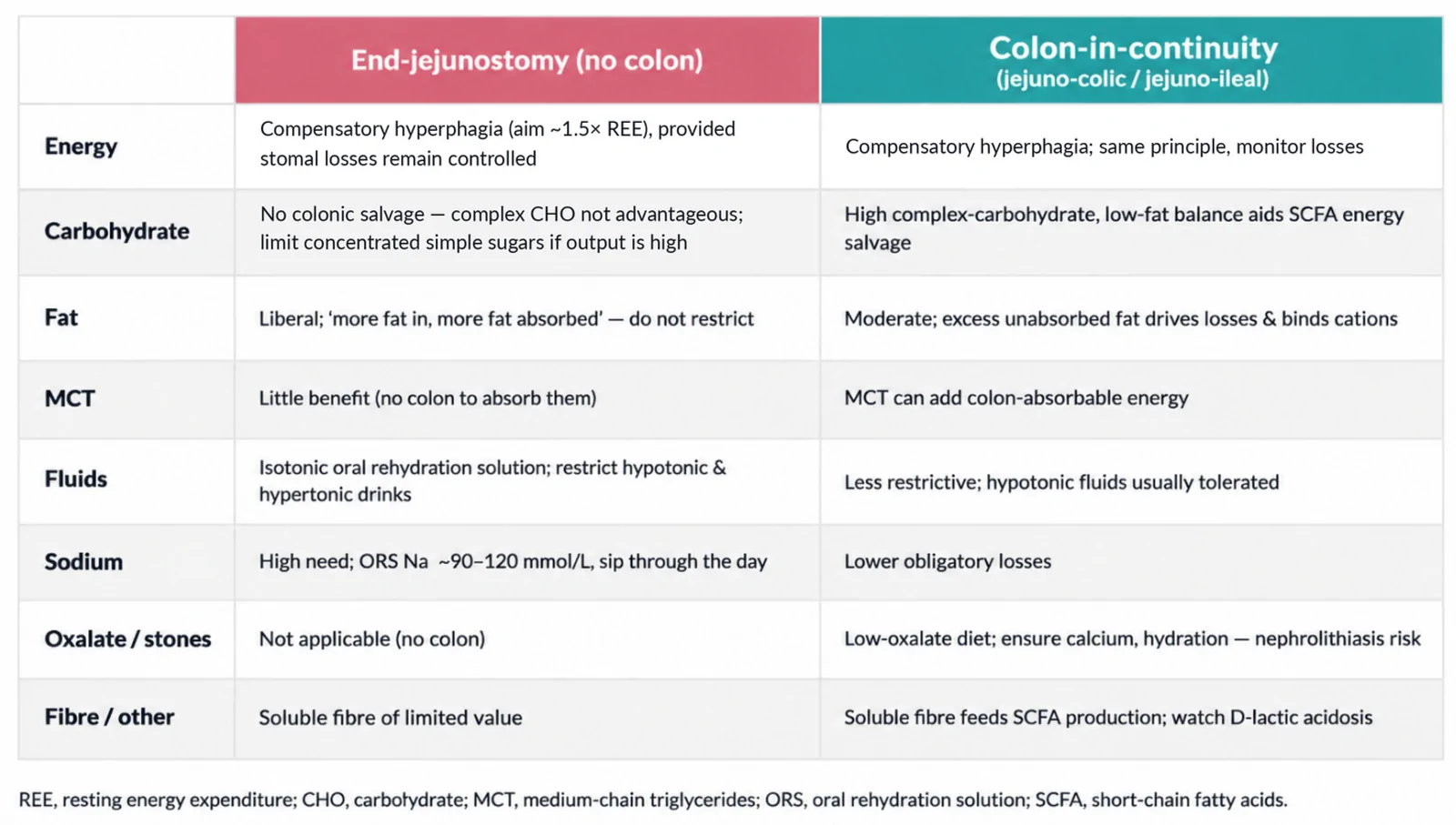
**Anatomy-tailored dietary strategy in short bowel syndrome**. The same lever (fat, carbohydrate, fluid or fibre) is pulled in opposite directions depending on whether the colon is in circuit. Energy intake through compensatory hyperphagia is encouraged in both phenotypes, provided that stomal or faecal losses remain controlled; in patients with an end-jejunostomy, simple sugars may require restriction. Original figure designed by the authors for this review; no part of it is reproduced or adapted from published guidelines or illustrations. The graphic was generated with the assistance of a generative artificial intelligence tool (Gemini 2.5 Pro, Google) from content and layout defined by the authors, who checked and edited the output and take full responsibility for its scientific accuracy.

**Figure 4:**
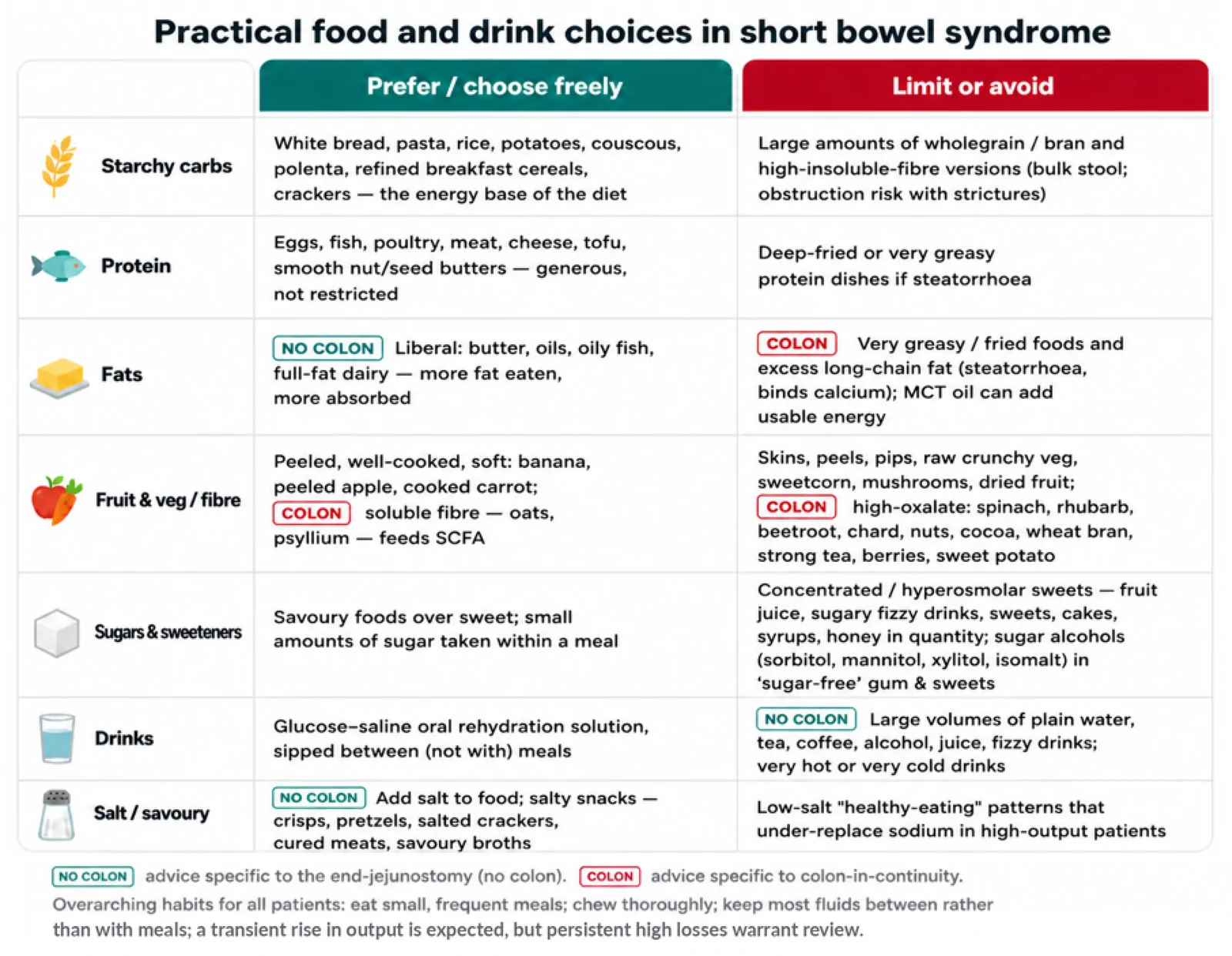
**Practical food and drink choices in short bowel syndrome.** A prefer-versus-avoid summary organised by food category, with anatomy-specific advice flagged for the end-jejunostomy (no colon) and colon-in-continuity phenotypes. It operationalises the physiology of Figure 1 and Figure 3 at the level of the plate. Original figure designed by the authors for this review; no part of it is reproduced or adapted from published guidelines or illustrations. The graphic was generated with the assistance of a generative artificial intelligence tool (Gemini 2.5 Pro, Google) from content and layout defined by the authors, who checked and edited the output and take full responsibility for its scientific accuracy.

**Figure 5:**
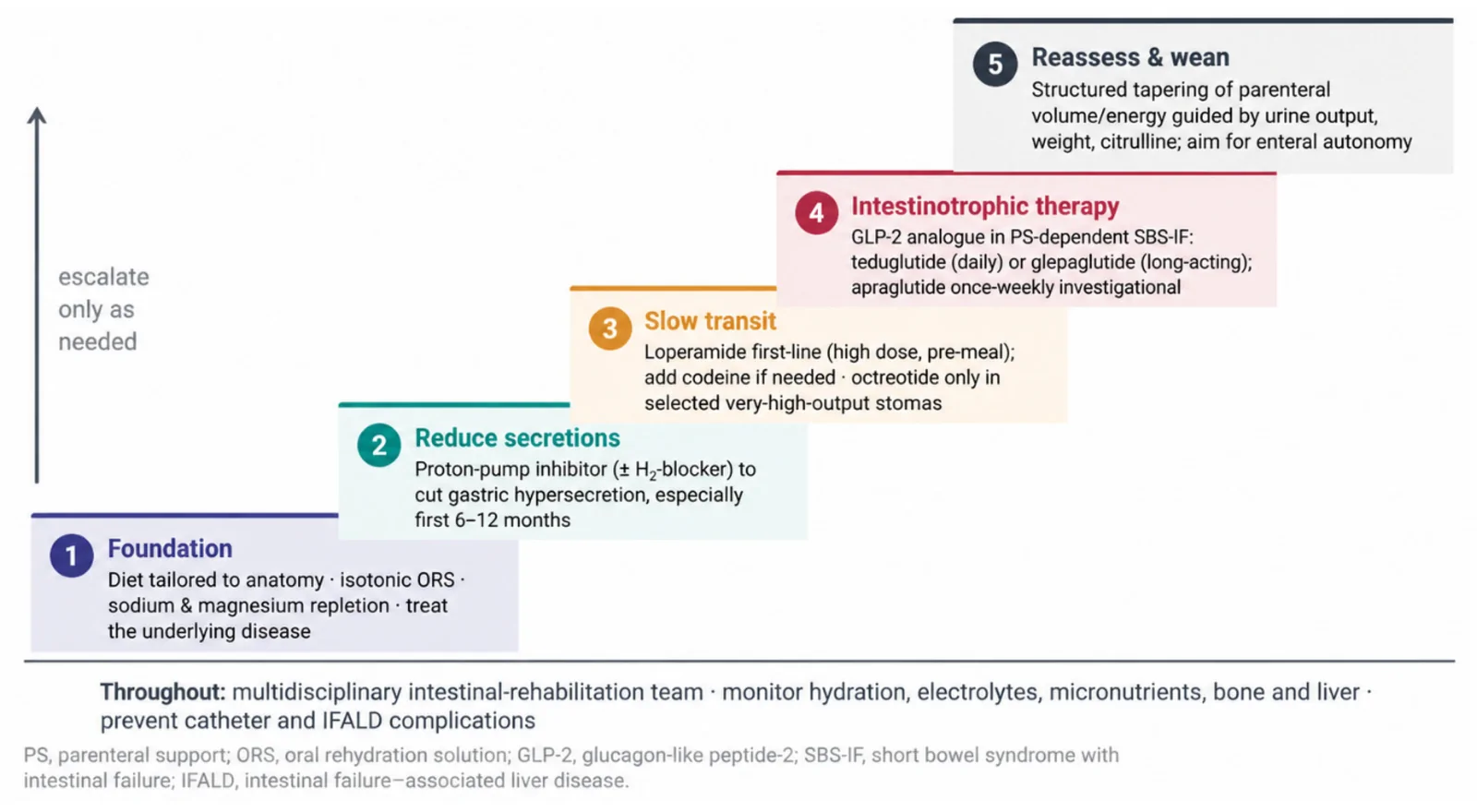
**A stepwise strategy to reduce parenteral support dependence.** Each rung is added only when the preceding measures are insufficient, and all sit on a foundation of anatomy-tailored diet, oral rehydration and electrolyte repletion. GLP-2 analogue therapy is the principal disease-modifying step in parenteral support-dependent patients; structured reassessment and weaning close the loop. Original figure designed by the authors for this review; no part of it is reproduced or adapted from published guidelines or illustrations. The graphic was generated with the assistance of a generative artificial intelligence tool (Gemini 2.5 Pro, Google) from content and layout defined by the authors, who checked and edited the output and take full responsibility for its scientific accuracy.

Because most recommendations in chronic intestinal failure rest on observational data and expert consensus, a formal grading exercise such as GRADE would convey a precision that a narrative synthesis of this literature cannot support. We therefore adopted a pragmatic alternative. Throughout the text, statements supported by randomised controlled trials are identified as such; recommendations issued by international guidelines are cited with their source; and practice points derived from observational series or expert consensus are presented explicitly as such. Table 1 maps the principal management domains to the nature of the evidence that supports them, separating formally issued guideline recommendations from statements of good practice. Generative artificial intelligence was used to assist in producing the graphics for Figure 1, Figure 2, Figure 3, Figure 4 and Figure 5, as declared in each figure legend; the authors verified all content and take full responsibility for it.

## 3. Pathophysiology That Drives Nutritional Decisions

### 3.1. Residual Anatomy Defines the Phenotype

Three residual anatomies account for the great majority of adult SBS and behave so differently that they are best regarded as distinct clinical entities (Figure 1) [5,29]. In the end-jejunostomy configuration, the jejunum ends in a stoma with no colon in continuity. These patients are typically “net secretors” because the jejunal mucosa is freely permeable to water and sodium; hypotonic or hypertonic oral intake draws fluid into the lumen, so that stomal output can exceed oral intake [30]. They lose large volumes of water, sodium, and magnesium, adapt little over time, and are the most PS-dependent group [5,31].

In the jejuno-colic configuration, the jejunum is anastomosed to the colon with little or no ileum remaining. Here, the colon becomes a digestive organ: unabsorbed carbohydrate is fermented by the microbiota to short-chain fatty acids (SCFA), salvaging up to ~1000 kcal/day, while colonic contact slows transit through the peptide-YY/GLP-2 “ileo-colonic brake” [31,32,33]. Colonic preservation reduces PS requirements but introduces two anatomy-specific hazards: calcium oxalate nephrolithiasis and D-lactic acidosis [31]. The jejuno-ileal configuration retains more than ~10 cm of terminal ileum plus the colon; with the ileal brake and bile salt/B12 handling preserved, this group has the best absorptive and adaptive capacity and rarely needs long-term support [5].

### 3.2. Adaptation Is a Moving Target

The remnant intestine changes over time. Three phases are conventionally described (Figure 2) [34]. The acute/hypersecretory phase (first three to four weeks) is dominated by massive fluid and electrolyte losses and gastric acid hypersecretion; PS is life-saving, and the priorities are haemodynamic and electrolyte stabilisation rather than nutritional “repletion.” The adaptation phase (weeks to one to two years) brings structural and functional remodelling (villus hypertrophy, crypt deepening, slowed transit) together with spontaneous hyperphagia; this is the window in which early enteral feeding and an anatomy-tailored diet do most of their work. In the maintenance/stabilisation phase (more than one to two years), a new absorptive steady state is reached, and it becomes possible to distinguish patients who have achieved enteral autonomy from those with established CIF who will need long-term support or intestinotrophic therapy [34,35].

Two absorptive concepts underpin this trajectory. Intestinal insufficiency denotes malabsorption compensated by hyperphagia and adaptation without intravenous support, whereas intestinal failure denotes uncompensated malabsorption requiring PS; metabolic balance studies formally separate the two [36]. Adaptive hyperphagia is a physiological ally: most patients with SBS spontaneously eat 1.5–2 times their resting energy expenditure, and net absorption correlates far better with total intake than with the fraction absorbed [37]. This single observation, that eating more rather than eating “right” drives absorption, shapes much of dietary practice.

## 4. Goals, Energy Needs and Monitoring

The objectives of nutritional care are to correct and prevent malnutrition and dehydration, to preserve or restore fat-free mass and function, to maximise enteral autonomy, and to avoid the complications of both the disease and its treatment [3,4,38]. Energy requirements are usually estimated at ~25–35 kcal/kg/day, but because absorption is incomplete, the delivered target must exceed the absorbed target; encouraging hyperphagia rather than imposing restriction is the guiding principle, and dietary counselling should be individualised and repeated as the patient adapts [6,37,39].

Objective monitoring anchors decisions. Beyond weight, hydration, and stomal/faecal output charts, plasma citrulline, an enterocyte-derived amino acid, provides a quantitative biomarker of residual absorptive mucosal mass; concentrations below ~20 µmol/L correlate with permanent intestinal failure and a low probability of PS independence [40]. Regular assessment of fluid balance (including a spot urine sodium level <20 mmol/L as an early marker of sodium depletion), renal function, magnesium, and fat-soluble and other micronutrients allows problems to be pre-empted rather than discovered late [4,5].

## 5. Dietary (Oral and Enteral) Management

### 5.1. General Principles

Oral diet remains first-line therapy and should be maintained or reintroduced as early as safely possible, because luminal nutrients are themselves the strongest stimulus to adaptation [34,39]. Practical measures that apply across anatomies include eating frequent small meals, separating solids from large volumes of fluid, chewing well, and encouraging a generous overall intake, since net absorption rises with the amount eaten. This encouragement nonetheless requires clinical judgement. Larger intakes are accompanied by a rise in stomal or faecal output, and in some patients, particularly those with an end-jejunostomy and a high-output stoma, unrestrained intake of rapidly fermentable simple sugars and of hypotonic or hyperosmolar fluids can drive output to levels that cause dehydration, electrolyte disturbances and peristomal skin erosion. In these circumstances, a temporary, dietitian-supervised restriction or modification of intake, most often of simple sugars and free fluids, is appropriate, and hyperphagia should be promoted only insofar as losses remain controlled and replaceable. Diet must then be tailored to the residual anatomy (Figure 3), because the same advice can help one phenotype and harm another [5,6]. In patients with SBS at risk of malnutrition and concomitant hyporexia, high-energy, high-protein oral nutritional supplements (ONS) may represent a useful adjunct to oral feeding in order to support adequate nutrient intake; however, their use should be individualised, with particular attention to the mode of administration and the osmolarity of the formulation. Thus, iso-osmolar formulations are generally preferred, as they may enhance intestinal absorption while reducing fluid losses [4].

### 5.2. The Patient with an End-Jejunostomy (No Colon)

These patients are defined by their fluid and sodium physiology. Hypotonic drinks (water, tea, alcohol, dilute juices) and hypertonic drinks paradoxically increase stomal output by driving net jejunal secretion; the cornerstone of therapy is therefore to restrict free hypotonic fluids and replace them with a glucose–saline oral rehydration solution (ORS) whose sodium concentration is high enough (~90–120 mmol/L) to exploit sodium–glucose co-transport and reverse net secretion [5,6,11,12]. In a randomised crossover balance study in patients with a high-output jejunostomy, sodium given as a sipped isotonic glucose–electrolyte solution kept every patient in positive sodium balance, whereas sodium chloride capsules and a glucose polymer solution left some patients in negative balance [12]. Sodium losses are large and must be replaced generously. Contrary to older teaching, these patients do not benefit from fat restriction: they absorb more fat when they eat more, so an energy-dense, unrestricted fat, higher-salt diet with a separate ORS is usually optimal [5,6,41]. This advice derives from metabolic balance studies: in patients with a jejunostomy or a short remnant small bowel, diets differing widely in fat content did not materially alter stomal output or overall energy absorption [8,9], and in a randomised crossover comparison of isocaloric high-carbohydrate and high-fat diets, energy absorption in patients without a colon did not depend on the macronutrient ratio, whereas the high-carbohydrate diet increased stomal output [32]. In concrete terms, this means encouraging salty, savoury, energy-dense foods (for example, crisps, salted crackers and pretzels, cheese, cured meats, oily fish, eggs, and butter or oil added freely to starchy staples such as bread, pasta, rice, and potatoes), while adding salt at the table and avoiding large sugary or hypotonic drinks with meals [6,41]. Restriction of concentrated simple sugars, including sugary drinks and confectionery taken in quantity, is often necessary in this phenotype, because their osmotic load draws fluid into the lumen and increases stomal losses [5,41]. Macronutrient composition otherwise matters relatively little because there is no colon to salvage malabsorbed substrate.

### 5.3. The Patient with Colon in Continuity

When the colon is preserved, the dietary calculus changes. A higher-complex-carbohydrate, moderate-fat diet is generally advised: unabsorbed carbohydrate is fermented to SCFA and salvaged as energy, whereas a large unabsorbed fat load is fermented less efficiently, binds divalent cations, and worsens diarrhoea and steatorrhoea [31,32]. In the randomised crossover study cited above, the high-carbohydrate diet increased energy absorption from 49% to 69% in patients with a preserved colon without increasing faecal volume [32], and in a series of 148 patients, the colon contributed up to approximately 4.2 MJ (about 1000 kcal) per day of salvaged energy as small bowel failure progressed [33]. Medium-chain triglycerides (MCT) can add colon-absorbable energy in this group specifically: in a randomised crossover balance study, replacing half of dietary long-chain fat with MCT increased overall energy absorption from 46% to 58% in patients with a colon, with no net energy gain in those without a colon [10]. Soluble fibre supports SCFA production. Fluid handling is less demanding than in the patient with a jejunostomy, and hypotonic fluids are usually tolerated [6].

In practice, the plate is built on starchy complex carbohydrates (bread, pasta, rice, potatoes, couscous, polenta, and refined cereals), with protein foods and only moderate fat; oats or psyllium are practical sources of soluble fibre, and MCT oil can be added when weight gain is difficult [6,32,41]. Very greasy, deep-fried, and high-long-chain-fat foods are limited, not least because unabsorbed fat promotes oxalate absorption [41].

Two colon-specific complications require dietary attention. Enteric hyperoxaluria and calcium oxalate stones arise because unabsorbed fatty acids bind calcium, leaving free oxalate to be absorbed by the colon; a low-oxalate diet, adequate calcium taken with meals, and good hydration reduce the risk [31,41]. Foods particularly high in oxalate (spinach, rhubarb, beetroot, chard, nuts and nut products, soy, cocoa and chocolate, wheat bran, strong tea, many berries, and sweet potato) are the ones to limit in this group [41]. D-lactic acidosis, a rare encephalopathy caused by colonic bacterial over-fermentation of carbohydrate to D-lactate, should be considered in a patient with a colon in continuity who presents with neurological symptoms and a high anion gap acidosis, and is managed by reducing rapidly fermentable carbohydrate and, at times, antibiotics [5,42].

The same principles extend to the jejuno-ileal configuration: with the terminal ileum and colon preserved, absorptive and adaptive capacity is largely intact, and a normal, energy-dense diet following the general measures outlined above is usually sufficient, without the specific restrictions required by the other two phenotypes; vitamin B12 status should nonetheless be monitored when a substantial length of ileum has been resected [5,6].

### 5.4. Foods and Drinks: What to Prefer and What to Limit

Translating these principles into everyday choices helps patients most (Figure 4). Across both phenotypes, the diet is built on starchy complex carbohydrates (bread, pasta, rice, potatoes, couscous, refined cereals) with generous protein (eggs, fish, poultry, meat, cheese, tofu, smooth nut butters), eaten as small, frequent meals with thorough chewing [6,39,41]. Fruit and vegetables are better tolerated peeled, well-cooked and soft (banana, peeled apple, cooked carrot) than raw and fibrous; skins, peels, pips, sweetcorn, mushrooms, whole nuts, seeds, and dried fruit tend to increase output and, with a narrowed anastomosis, pose an obstruction risk [41].

Several items are best limited or avoided in most patients: concentrated, hyperosmolar sweet foods, and drinks (fruit juice, sugary fizzy drinks, sweets, cakes, syrups, and honey in quantity), which draw fluid into the lumen; sugar alcohols (sorbitol, mannitol, xylitol, isomalt) hidden in ‘sugar-free’ gum, sweets and cough drops, which act as osmotic laxatives; and excess alcohol and caffeine, which stimulate the gut and act as diuretics [41]. The remaining choices are then tailored by anatomy, following the phenotype-specific strategies detailed in Section 5.2 and Section 5.3 and summarised in Figure 3 and Figure 4 [5,6,41].

### 5.5. Fluid and Electrolyte Therapy

Dehydration and sodium depletion, rather than calorie deficit, are the most common reasons for hospital readmission and for failure to wean from PS [5,6].

To safely absorb water and sodium enterally, fluid replacement must exploit the active, coupled transport of sodium and glucose via the sodium glucose cotransporter 1 (SGLT-1) on the jejunal brush border [4,5,6]. The active intracellular transport of sodium and glucose generates a localised osmotic gradient, drawing water passively into the enterocytes via solvent drag [4,5].

The practical toolkit is: limit hypotonic and hypertonic drinks; provide a glucose–saline ORS sipped throughout the day; replace magnesium (often as an evening oral dose, sometimes parenterally when depletion is severe); and use a spot urine sodium level of <20 mmol/L as an early warning of total-body sodium depletion, which itself aggravates magnesium loss through secondary hyperaldosteronism [5]. Some patients with a jejunostomy ultimately require subcutaneous or intravenous saline even when caloric needs are met enterally.

### 5.6. Macronutrients and Micronutrients

Energy and protein. The diet should be energy-dense; protein is generally well tolerated and does not need restriction, and whole-protein diets are as well absorbed as peptide-based formulas in most patients [6,39]. Fat and MCT are handled as described in Section 5.2 and Section 5.3. Carbohydrate and fibre are leveraged for colonic salvage only when the colon is in continuity.

Micronutrient deficiencies are common in SBS and require structured monitoring at least once per year, with parenteral or oral supplementation tailored to laboratory values (Table 2) [4,6]. Water-soluble vitamins are absorbed in the proximal small bowel, and deficiencies are rare, with the critical exception of Vitamin B12 [4]. Resection of more than 60 to 100 cm of terminal ileum completely abolishes active B12-intrinsic factor absorption, necessitating life-long monthly parenteral injections of 300 to 1000 µg [4,5,6]. Fat-soluble vitamins (A, D, E, K) are highly susceptible to malabsorption due to a depleted bile acid pool resulting from ileal resection [4,5]. Vitamin D deficiency is highly prevalent and actively drives metabolic bone disease (MBD) [4]. Standard intravenous lipid emulsions provide phylloquinone (Vitamin K), but additional supplementation may be required in distal bowel disease or in patients on lipid-free PN [4]. High doses of Vitamin C (>200 mg/day) in HPN formulations can increase urinary oxalate excretion, elevating nephrolithiasis risk [4].

Enteral or, where absorption is inadequate, parenteral micronutrient provision should be guided by regular measurement rather than assumption [3,4].

Trace element management is critical in patients on long-term HPN. Zinc is excreted in large quantities in diarrhoeal and stomal fluids, and patients with high-output fistulas or stomas may require up to 12 to 17 mg of intravenous zinc per litre of fluid lost [4]. Selenium deficiency is common and associated with an increased risk of serious infections [4]. Copper should be monitored closely and restricted in cholestasis, as it is primarily excreted in bile, and toxicity can lead to severe hepatic accumulation [4]. Chromium is present as a widespread contaminant in parenteral components, and excess chromium accumulates in tissues and may cause renal impairment [4]. Manganese toxicity represents a major clinical concern in long-term HPN. Fixed-dose multi-trace element solutions often deliver manganese levels five to six times above current daily requirements (~55 µg/day), leading to manganese deposition in the globus pallidus, presenting as severe parkinsonian-like neurotoxicity [4]. Iron deficiency is highly prevalent, affecting 30–50% of patients on HPN and requiring regular intravenous iron infusions or custom HPN compounding [4]. Metabolic bone disease (MBD), with reduced bone mineral density documented in 84% of patients on HPN and osteoporosis in 41% in a European multicentre cross-sectional survey [43], is multifactorial, driven by chronic inflammation, corticosteroid use, and PN-related hypercalciuria [4]. Monitoring via repeated DXA scans is recommended, and management includes optimising calcium, magnesium, and phosphate ratios in the PN bag, followed by intravenous bisphosphonates (clodronate, pamidronate) or subcutaneous denosumab [4].

## 6. Pharmacological Adjuncts That Spare Parenteral Support

Drug therapy in SBS is best conceived as a stepwise escalation layered on top of diet and fluids, each step reserved for patients not controlled by the previous one (Figure 5) [4,5]. Antisecretory therapy with a proton pump inhibitor reduces the gastric acid hypersecretion that magnifies early fluid losses [13] and is especially useful in the first 6–12 months. Antimotility agents slow transit and reduce output: high-dose loperamide taken before meals is first-line (it undergoes enterohepatic recirculation that is lost in SBS, so doses higher than usual are often needed), with codeine phosphate added when necessary. Octreotide reduces very high stomal output but is reserved for selected refractory cases because it may impair adaptation and carries its own risks [5,6].

A major therapeutic advance is GLP-2 analogue therapy, which directly targets adaptation (Table 3). GLP-2 is an intestinotrophic hormone that increases villus height and crypt depth, slows transit, and reduces secretion, thereby improving absorption [44]. Teduglutide, a DPP-IV-resistant GLP-2 analogue, reduced PS requirements versus placebo in randomised phase 3 trials: in the pivotal STEPS study, 63% of patients treated with teduglutide achieved a 20–100% reduction in PS volume versus 30% on placebo, with rising plasma citrulline and, in extension studies, progressive weaning and enteral autonomy in a subset [14,15,45]. Response is more likely in patients with lower baseline PS requirements and non-inflammatory aetiology [46]. Longer-acting analogues designed to ease treatment burden have followed: glepaglutide, which reduced faecal output and increased intestinal absorption in a phase 2 crossover trial [47], significantly reduced weekly PS volume versus placebo when given twice weekly in the phase 3 EASE-1 trial (mean reduction of 5.13 versus 2.85 L/week), whereas once-weekly dosing did not reach statistical significance [16]. Apraglutide, a once-weekly analogue, improved intestinal fluid absorption in a randomised phase 2 study [17] and met its primary endpoint of reduced weekly PS volume in the global phase 3 STARS trial (NCT04627025), with a relative reduction of 25.5% versus 12.5% on placebo at week 24 [18,48]; the trial stratified patients by stoma versus colon-in-continuity anatomy, and results from its extension study suggest sustained reductions in PS, with enteral autonomy in some patients [49]. The complete phase 3 results have so far been reported only as congress abstracts. GLP-2 therapy should be initiated within an experienced intestinal rehabilitation programme, with diet and PS actively re-titrated as absorption improves.

## 7. Parenteral Nutrition and Home Parenteral Nutrition

### 7.1. Indications and Composition

Parenteral support is indicated whenever enteral intake plus adaptation cannot maintain hydration, electrolyte balance, and nutritional status, which is the defining feature of intestinal failure [1,3]. In stable CIF, PS is delivered as home parenteral nutrition (HPN), which is the primary, life-preserving therapy and is more cost-effective than prolonged hospitalisation [19]. The admixture is individualised to the patient’s measured deficits rather than to standardised formulae: energy is provided as a glucose–lipid mixture, with amino acids to meet protein needs, and with sodium, potassium, magnesium, calcium, phosphate, trace elements, and vitamins titrated to serial biochemistry [3,19]. In practice the admixture can be delivered as an individually compounded bag prepared by a hospital or commercial pharmacy, as a commercial ready-to-use multi-chamber bag (MCB), or as a hybrid regimen in which an MCB is supplemented with electrolytes, vitamins, and trace elements, or combined with separate saline infusions [19,20]. Compounded bags allow full individualisation and are usually required when fluid and electrolyte needs are high, as in patients with an end-jejunostomy; MCBs are simpler to supply, have a longer shelf life, and require less preparation time, but their fixed composition rarely matches the needs of patients with SBS without additions. Systematic reviews of largely observational comparisons suggest that MCBs are well tolerated and may be associated with fewer bloodstream infections and lower costs in hospital settings, whereas evidence in the home setting remains limited [21,22]. Many patients with a jejunostomy need proportionally more intravenous fluid, sodium, and magnesium than calories: it is a saline-and-magnesium problem as much as a feeding problem.

The standard daily macronutrient composition of an HPN formula should be tailored on the basis of formal nutritional assessments (Table 4). Energy requirements generally range from 20 to 35 kcal/kg/day (derived from glucose and lipids), and protein requirements range from 0.8 to 1.4 g of L-amino acids/kg/day [4]. Water and electrolyte requirements must be adjusted based on stomal and faecal outputs, typically providing 25 to 35 mL of water/kg/day, 1.0 to 1.5 mmol of sodium/kg/day, and 1.0 to 1.5 mmol of potassium/kg/day [4]. Tight glycaemic control is targeted during infusion, maintaining blood glucose below 180 mg/dL (10.0 mmol/L) and a target HbA1c of <7% in patients with diabetes [4]. For safe, long-term HPN delivery, vascular access must be managed by an experienced nutrition support team (NST). Tunnelled central venous catheters (CVCs) or subcutaneously anchored catheters are the gold standard [4,6]. Access to the superior vena cava via the right internal jugular or subclavian vein is strongly preferred to reduce local thrombosis risk. To prevent life-threatening catheter-related bloodstream infections (CRBSI), strict aseptic non-touch techniques must be maintained [4,5].

### 7.2. Lipid Emulsions and Intestinal Failure–Associated Liver Disease

Lipid emulsions supply energy and essential fatty acids but are also a modifiable risk factor for intestinal failure–associated liver disease (IFALD), a severe, progressive, and potentially life-threatening hepatobiliary complication of long-term home parenteral nutrition, ranging from steatosis and cholestasis to fibrosis [4,51,52]. In a prospective cohort of 90 adults with permanent intestinal failure on HPN, chronic cholestasis developed in 65% and complicated liver disease in 41.5%, and a parenteral lipid intake of 1 g/kg/day or more was among the factors associated with liver disease [24]. In adults, IFALD is a multifactorial condition driven by both patient-specific and PN-associated factors [4]. Patient-specific risks include severe small bowel resection (remnant <50–100 cm), loss of the colon in continuity, recurrent central line sepsis, and chronic enteral starvation [4,53]. Enteral starvation impairs the physiological secretion of gut-derived hepatoprotective hormones (such as gastrin, secretin, and cholecystokinin), causing gallbladder stasis, biliary sludge accumulation, and cholestasis [53]. Sepsis acts as a powerful trigger: circulating lipopolysaccharides (LPS) and endotoxins translocate from the gut lumen through a compromised, hyperpermeable epithelial barrier into the portal circulation, activating hepatic Kupffer cells and unleashing a cascade of pro-inflammatory cytokines (TNF-α, IL-6) that drive cholestasis and progressive fibrosis [4,53].

To prevent and treat IFALD, several lipid modification and clinical strategies should be implemented (Table 5):

- **Avoid or limit soybean oil emulsion:** For long-term HPN (more than six months), pure soybean oil-based (n-6-rich) lipid emulsions should generally be avoided as the sole lipid source and, when used, limited to less than 1.0 g/kg/day, since higher doses are associated with IFALD [24]; overall lipid infusion of 0.8–1.5 g/kg/day is safe and should not exceed 2.6 g/kg/day [3,4].
- **Use alternative mixed emulsions:** If total lipid requirements exceed 1.0 g/kg/day, composite emulsions containing olive oil, medium-chain triglycerides (MCT) and/or fish oil, with a more favourable n-6:n-3 ratio, should be preferred. These formulations reduce the total omega-6 and phytosterol load and provide high concentrations of α-tocopherol, exerting a strong antioxidant and hepatoprotective effect [3,51]. Randomised comparative trials in adults on long-term PN support this choice, although they were small and used biochemical rather than clinical endpoints: an olive-oil-based emulsion was as well tolerated as a soybean oil emulsion over three months of HPN [25]; a soybean/MCT/olive/fish oil emulsion produced lower transaminase and bilirubin concentrations than a pure soybean oil emulsion after four weeks [26]; and an n-3-enriched MCT/LCT emulsion proved safe and well tolerated in the multicentre, double-blind HOME trial [27]. A recent international expert consensus on lipid emulsions in long-term HPN reached the same conclusion [23].
- **Administer pure fish oil emulsions in established cholestasis:** In patients with cholestasis or hyperbilirubinaemia, pure fish oil-based emulsions (rich in omega-3 fatty acids) or mixed lipid emulsions can successfully reverse cholestasis, normalise transaminases, and arrest progressive fibrosis [4,52]. Omega-3 fatty acids downregulate pro-inflammatory pathways and promote fatty acid oxidation via PPAR-α activation.
- **Implement cyclic infusion:** Cycling the PN infusion over 10 to 14 h overnight, instead of 24 h continuous administration, is strongly recommended [4,6]. Cyclic infusion allows the liver a ‘fasting interval’, promoting mobilisation of fatty acids from the liver and decreasing steatosis [53].
- **Optimise enteral/oral feeding:** Even minimal, trophic oral or enteral intake should be encouraged to stimulate gallbladder contraction and biliary flow, reducing the risk of cholelithiasis and cholestasis [19,51].

Prevention of IFALD also depends on avoiding overfeeding and glucose overload, treating catheter sepsis promptly, and preventing small bowel bacterial overgrowth [19,51]. In patients with double enterostomies, chyme reinfusion (re-feeding enteroclysis) successfully restores the bile salt–FGF19–FXR axis, markedly improving liver function and allowing HPN weaning [4,53,54].

**Table 5:** Lipid emulsion strategy and other measures for the prevention and treatment of intestinal failure–associated liver disease [3,4,19,51,53].

Strategy	Practical Rule	Rationale
Avoid or limit pure soybean oil emulsion	Avoid as the sole lipid source; if used, below 1.0 g/kg/day for HPN beyond 6 months; total lipid 0.8–1.5 g/kg/day, never above 2.6 g/kg/day	High n-6 fatty acid and phytosterol load promotes IFALD; lipid intake of 1 g/kg/day or more was associated with chronic cholestasis in a prospective HPN cohort [24]
Prefer composite emulsions	Olive oil, MCT, and/or fish oil mixtures when lipid requirements exceed 1.0 g/kg/day	More favourable n-6:n-3 ratio; α-tocopherol content with antioxidant, and hepatoprotective effects; lower transaminases and bilirubin levelsthan with soybean oil in a randomised trial [26]; comparable tolerance of olive oil and n-3-enriched emulsions in randomised trials [25,27]
Pure fish oil emulsions	In established cholestasis or hyperbilirubinaemia	Omega-3 fatty acids downregulate pro-inflammatory pathways and promote fatty acid oxidation via PPAR-α; these emulsions can reverse cholestasis
Cyclic infusion	10–14 h overnight rather than continuous administration	Hepatic fasting interval promotes mobilisation of fatty acids and reduces steatosis
Trophic enteral or oral intake	Encourage even minimal intake	Stimulates gallbladder contraction and biliary flow, reducing sludge, cholelithiasis and cholestasis
General measures	Avoid overfeeding and glucose overload; treat catheter sepsis promptly; prevent small bowel bacterial overgrowth	Removes co-factors of hepatic injury, including endotoxin-driven Kupffer cell activation
Chyme reinfusion	In patients with double enterostomies	Restores the bile salt–FGF19–FXR axis, improving liver function and facilitating HPN weaning

### 7.3. Weaning and the Drive Toward Enteral Autonomy

Whenever adaptation, diet, and pharmacotherapy improve absorption, PS should be actively tapered rather than left unchanged. Weaning is guided by objective targets (stable weight and hydration, adequate urine output and sodium, and rising citrulline) and typically proceeds by reducing infused volume and energy in structured steps, often first by dropping infusion nights per week before reducing per-session volume [4,46]. Roughly half of adults with SBS regain enteral autonomy over time, most within the first two years, with the probability being higher with greater residual small bowel length, preserved colon, and higher citrulline [35,55]. The remainder have established CIF and are candidates for long-term HPN and GLP-2 therapy [3,55]. Surgery retains a defined place in this therapeutic pathway: restoration of intestinal continuity to recruit distal unused bowel and, in selected patients, autologous reconstructive (bowel-lengthening) procedures can improve absorptive capacity and facilitate weaning [56]. Intestinal transplantation remains a life-saving option for patients with irreversible CIF who develop life-threatening complications of HPN, such as IFALD, progressive loss of central venous access, or recurrent catheter-related sepsis, and candidacy should be assessed in a dedicated transplant centre [3,57].

### 7.4. Complications and Long-Term Surveillance

Long-term PS carries a defined complication burden that must be actively surveilled [3,19]. Catheter-related bloodstream infection is the most frequent serious complication and is minimised by strict aseptic technique, appropriate central venous access device selection, and, in recurrent cases, taurolidine or ethanol catheter locks [58]; catheter occlusion and central vein thrombosis are prevented by careful line care and tip positioning. IFALD and metabolic bone disease require periodic hepatic and skeletal assessment. A structured monitoring schedule (clinical, anthropometric and biochemical) conducted by an experienced team underpins both safety and quality of life, and patients benefit from education and peer support networks [3,19,59].

## 8. Clinical Outcomes Across Nutritional Support Strategies

The endpoints that matter most to patients and clinicians are the achievement of enteral autonomy, survival, the complication burden of long-term parenteral support, and quality of life. Enteral autonomy is regained by roughly half of adults with SBS, most within the first two years, and its probability increases with residual small bowel length, colonic preservation and higher plasma citrulline [35,40,55]. In a multicentre European and North American cohort of 472 patients starting HPN for benign chronic intestinal failure, 36.5% were weaned from parenteral support within five years, with four fifths of them being weaned during the first year [60]; a large single-centre series of 268 patients with non-malignant SBS likewise identified residual anatomy as the dominant determinant of lasting HPN dependence [61].

Survival on HPN is determined mainly by age and by the underlying disease rather than by parenteral support itself. In the same multicentre cohort, overall survival was 88%, 74% and 64% at one, three, and five years, and most deaths occurred within the first years of treatment [60]. HPN therefore remains the life-preserving standard of care in chronic intestinal failure [3,19], with catheter-related bloodstream infection, central vein thrombosis, IFALD, and metabolic bone disease as the principal treatment-related determinants of morbidity (Section 7.2 and Section 7.4).

Among the interventions reviewed here, only GLP-2 analogues have been tested against placebo in adequately powered randomised trials. Teduglutide increased the proportion of patients achieving a 20–100% reduction in parenteral support volume (63% versus 30%) [14,15], with progressive weaning in extension studies [45], and reductions in parenteral support were accompanied by measurable gains in disease-specific quality of life [50]; phase 3 data for glepaglutide and apraglutide point in the same direction [16,17,18,48]. By contrast, dietary strategies, oral rehydration solutions, and antisecretory or antimotility drugs rest on metabolic balance studies, small crossover trials, and observational series; their effect on hard outcomes has not been tested in randomised trials, and comparisons of outcomes across nutritional support strategies therefore remain indirect (Table 1).

## 9. Organisation of Care

Outcomes in SBS-IF are demonstrably better when patients are managed by a coordinated, multidisciplinary intestinal-rehabilitation team (gastroenterologist, surgeon, specialist dietitian, nutrition nurse, and pharmacist), with early referral of complex patients to centres with expertise in both medical and surgical treatment [4,28]. Such teams integrate the elements described above (anatomy-tailored diet, fluid and electrolyte optimisation, antisecretory and antimotility drugs, GLP-2 therapy, and safe HPN) and are best placed to maximise the opportunity for weaning, to prevent HPN-related complications, and to assess candidacy for transplantation when HPN fails [3,28].

## 10. Key Messages for Practice

However complex an individual case may appear, the management of short bowel syndrome converges on a small number of principles that, applied consistently, most reliably improve outcomes at the bedside. Box 1 distils the preceding sections into a quick reference for the whole multidisciplinary team: gastroenterologist, surgeon, dietitian, nutrition nurse and pharmacist alike. Each point is developed in the relevant section above and is intended as a practical aide-mémoire rather than a substitute for individualised assessment.

Box 1 **Key messages for practice.**

- **Classify first.** Establish the residual anatomy (end-jejunostomy vs colon-in-continuity vs jejuno-ileal) and the adaptation phase before giving any dietary advice: they change the advice completely.
- **Feed generously, monitor losses.** Compensatory hyperphagia drives absorption and should generally be encouraged; an energy-dense diet with individualised counselling is first-line in every phenotype, with intake modulated when stomal or faecal losses become excessive.
- **Tailor the diet to the colon.** No colon: liberal fat, high-sodium ORS, restrict hypotonic fluids. Colon present: higher complex-carbohydrate/moderate-fat, MCT useful, watch oxalate and D-lactic acidosis.
- **Master fluids and sodium.** Dehydration and sodium depletion are the leading causes of readmission and failed weaning; a glucose–saline ORS and magnesium repletion are essential tools.
- **Escalate drugs stepwise.** PPI → high-dose loperamide ± codeine → (selected) octreotide → GLP-2 analogue in PS-dependent SBS-IF.
- **Use PS wisely and safely.** Individualise the admixture, avoid pure soybean oil lipid or cap it below 1 g/kg/day and prefer composite emulsions to limit IFALD, and taper PS actively toward enteral autonomy.
- **Refer early to a specialist team.** Multidisciplinary intestinal rehabilitation improves weaning rates, prevents complications and identifies transplant candidates.

## 11. Future Perspectives

Several areas of uncertainty deserve priority in future research. Dietary recommendations in SBS derive largely from metabolic balance studies conducted decades ago in small numbers of patients; randomised trials comparing anatomy-tailored dietary strategies, oral rehydration formulations, and structured counselling models against usual care, with enteral autonomy and quality of life as endpoints, are feasible within collaborative networks and have not been performed. The contribution of the gut microbiome is a second open field: colonic energy salvage, D-lactic acidosis, and possibly intestinal adaptation itself are microbially mediated. Profiling studies in adults with SBS have shown a dysbiotic faecal and mucosa-associated microbiota dominated by *Lactobacillus* and depleted of strict anaerobes [62,63]; faecal lactate accumulation, and in particular a high faecal D/L-lactate ratio, identified the patients at risk of D-lactic encephalopathy [42], and transfer of this microbiota to germ-free rats suggested a contribution to post-resection adaptation [64]. It remains unknown whether the microbiome can be modulated to therapeutic advantage in SBS. Third, plasma citrulline remains the only widely used biomarker of absorptive mucosal mass, and better predictors of weaning and response to intestinotrophic therapy are needed.

With three GLP-2 analogues now supported by randomised evidence, the personalisation of intestinotrophic treatment has become a concrete question: predictors of response, criteria for starting and stopping therapy, anatomy-specific dosing, long-term safety, including neoplasia surveillance, and cost-effectiveness all require prospective study, ideally within international registries that also capture patient-reported outcomes. Finally, the place of surgical rehabilitation, chyme reinfusion, and, on a longer horizon, tissue-engineered intestine should be defined against this evolving pharmacological background.

## 12. Limitations

This article is a narrative review, and its limitations follow from that format. The literature search, although structured, was not systematic: no protocol was registered, screening was not performed in duplicate, and a formal assessment of risk of bias was not undertaken, so a degree of selection bias in the studies discussed cannot be excluded. No quantitative synthesis was attempted, and the strength of individual statements is indicated descriptively rather than through a formal grading system such as GRADE, which we considered unsuited to a body of evidence composed largely of observational studies and consensus recommendations. The underlying evidence itself carries limitations that no review format can overcome: most guidance in chronic intestinal failure rests on observational series and expert opinion, randomised data are essentially confined to GLP-2 analogues, and the marked heterogeneity of residual anatomies, underlying diseases and adaptation trajectories restricts the generalisability of findings from any single cohort. The practical syntheses offered in the figures, tables and key messages of this review reflect the authors’ interpretation of this evidence and should be applied with corresponding caution.

## 13. Conclusions

The management of short bowel syndrome is the management of absorption. By reading the two variables that matter most, namely what anatomy remains and where the patient sits on the adaptation curve, the clinician can deploy a coherent sequence of interventions: an anatomy-tailored diet that harnesses hyperphagia and, where possible, colonic energy salvage; a disciplined fluid, sodium, and magnesium strategy; a stepwise pharmacological ladder culminating in GLP-2 analogues; and a safely delivered, well-monitored parenteral programme with lipid strategies that protect the liver. Applied within an experienced multidisciplinary team, this integrated approach maximises enteral autonomy and quality of life while minimising the complications that have historically defined life with a short bowel.

## Figures and Tables

**Table 1:** Principal recommendations for the nutritional management of short bowel syndrome with intestinal failure in current international guidelines, according to the nature of the supporting evidence.

Management Domain	Recommendation and Guideline Source	Nature of Supporting Evidence
Definition and classification of intestinal failure	Requirement for intravenous supplementation defines intestinal failure; functional classification into types I–III (ESPEN [1,3,4])	Formal consensus recommendation; observational data
Dietary counselling	Anatomy-tailored dietary advice with encouragement of hyperphagia; individualised, repeated counselling (ESPEN [3,4])	Metabolic balance studies [8,9,10] and observational series; expert consensus
Fluid and electrolyte therapy	Restriction of hypotonic and hypertonic fluids with a glucose–saline oral rehydration solution in patients with an end-jejunostomy (ESPEN [3,4,6])	Small physiological studies [11,12]; expert consensus
Antisecretory and antimotility drugs	Proton pump inhibitor in the first months; loperamide as the first-line antimotility agent (ESPEN [3,4])	Small crossover trials [13] and observational data; expert consensus
GLP-2 analogues	Teduglutide in patients dependent on parenteral support despite optimised conventional therapy (ESPEN [4])	Randomised placebo-controlled phase 3 trials [14,15,16,17,18]
Composition and delivery of home parenteral nutrition	Individualised admixture, delivered as a pharmacy-compounded bag, a commercial multi-chamber bag (MCB) or a hybrid regimen (MCB with individual additions, or combined with separate intravenous saline); tunnelled or subcutaneously anchored central venous catheter; strict aseptic protocols (ESPEN [3,4,19,20])	Observational data; formal recommendation partly consensus-based; systematic reviews of observational comparisons of MCB and compounded bags [21,22]
Lipid strategy and IFALD	Pure soybean oil emulsion generally avoided as the sole lipid source or, when used, limited to below 1.0 g/kg/day in long-term home parenteral nutrition; composite emulsions containing olive oil, MCT and/or fish oil preferred (ESPEN [3,4]; expert consensus [23])	Observational studies [24], small randomised trials with biochemical endpoints [25,26,27] and pathophysiological rationale; expert consensus
Organisation of care	Management within multidisciplinary intestinal-rehabilitation programmes with early referral of complex patients (ESPEN [3,4,28])	Observational comparisons; good practice statement

**Table 2:** Micronutrient supplementation and monitoring in short bowel syndrome with intestinal failure [3,4,5,6].

Micronutrient	Clinical Issue in SBS-IF	Supplementation	Suggested Monitoring
Vitamin B12	Resection of more than 60–100 cm of terminal ileum abolishes active B12–intrinsic factor absorption	Parenteral 300–1000 µg monthly, lifelong	Serum B12 at least yearly
Vitamin D	Deficiency highly prevalent; drives metabolic bone disease	Oral or parenteral repletion guided by levels	25-OH vitamin D at least yearly; periodic DXA
Vitamins A and E	Malabsorbed when the bile-acid pool is depleted after ileal resection	Oral or parenteral repletion guided by levels	Serum levels at least yearly
Vitamin K	Provided by standard lipid emulsions; may be insufficient in distal bowel disease or lipid-free PN	Additional supplementation when indicated	INR; levels where available
Vitamin C	Doses above 200 mg/day in HPN increase urinary oxalate excretion	Avoid high doses, particularly in stone-formers	Review of HPN formulation
Zinc	Large losses in diarrhoeal and stomal fluid	Up to 12–17 mg intravenously per litre of fluid lost in high-output states	Serum zinc, interpreted with inflammatory markers
Selenium	Deficiency common; associated with serious infections	Intravenous supplementation in HPN	Serum selenium at least yearly
Copper	Excreted in bile; accumulates in cholestasis with hepatic toxicity	Restrict in cholestasis	Serum copper; liver biochemistry
Chromium	Widespread contaminant of parenteral components; tissue accumulation and possible renal impairment	Avoid routine excess	Periodic levels in long-term HPN
Manganese	Fixed-dose solutions may exceed requirements (~55 µg/day) 5- to 6-fold; deposition in the globus pallidus with Parkinsonian-like neurotoxicity	Reduce or omit in long-term HPN	Whole-blood manganese; neurological review
Iron	Deficiency in 30–50% of patients on HPN	Separate intravenous iron infusions or custom compounding	Ferritin and transferrin saturation

**Table 3:** GLP-2 and GLP-2 analogue therapy in short bowel syndrome with intestinal failure.

Agent	Regimen	Principal Clinical Evidence	Main Safety Considerations
Native GLP-2	Not used therapeutically	Physiological studies: increased villus height and crypt depth, slowed transit, and reduced secretion [44]	Rapid degradation by DPP-IV precludes therapeutic use
Teduglutide	0.05 mg/kg subcutaneously once daily	Phase 3 STEPS trial: 63% versus 30% of patients achieved a 20–100% reduction in parenteral support volume; progressive weaning and enteral autonomy in a subset during extension studies [14,15,45]; response predicted by lower baseline requirements and non-inflammatory aetiology [46]; reductions in parenteral support accompanied by quality-of-life gains [50]	Abdominal pain, nausea, stoma changes, and fluid shifts; intestinotrophic effect requires colonoscopic surveillance for gastrointestinal polyps before and during treatment
Glepaglutide	Subcutaneously once or twice weekly	Phase 2: reduced faecal output [47]; phase 3 EASE-1: significant reduction in parenteral support volume with twice-weekly dosing, not significant with once-weekly dosing [16]	Injection-site reactions; class effects as above
Apraglutide	Subcutaneously once weekly	Phase 2: improved intestinal fluid absorption [17]; phase 3 STARS trial (NCT04627025) met its primary endpoint of reduced weekly parenteral support volume (relative reduction of 25.5% versus 12.5% at week 24) [18,48], with stratification by stoma versus colon-in-continuity anatomy	Class effects as above; anatomy-specific dosing under evaluation

**Table 4:** Reference daily requirements for home parenteral nutrition in stable adults with short bowel syndrome and chronic intestinal failure [4,6].

Component	Usual Daily Requirement	Notes
Energy	20–35 kcal/kg	Glucose–lipid mixture; avoid overfeeding and glucose overload
Protein	0.8–1.4 g/kg of L-amino acids	Titrated to nutritional status and losses
Water	25–35 mL/kg	Increased according to stomal and faecal outputs
Sodium	1.0–1.5 mmol/kg	Requirements often higher in patients with an end-jejunostomy
Potassium	1.0–1.5 mmol/kg	Adjusted to serial biochemistry
Magnesium, calcium, phosphate	Titrated to serial biochemistry	Hypomagnesaemia frequent; ratios optimised for bone protection
Glycaemic targets	Blood glucose below 180 mg/dL (10.0 mmol/L) during infusion	Target HbA1c below 7% in patients with diabetes
Vascular access	Tunnelled central venous catheter or subcutaneously anchored catheter	Superior vena cava access via the right internal jugular or subclavian vein preferred; strict aseptic non-touch technique

## Data Availability

No new data were created or analysed in this study. Data sharing is not applicable to this article.

## References

[1] Pironi L., Arends J., Baxter J., Bozzetti F., Peláez R.B., Cuerda C., Forbes A., Gabe S., Gillanders L., Holst M. (2015). ESPEN endorsed recommendations. Definition and classification of intestinal failure in adults. Clin. Nutr..

[2] O’Keefe S.J.D., Buchman A.L., Fishbein T.M., Jeejeebhoy K.N., Jeppesen P.B., Shaffer J. (2006). Short bowel syndrome and intestinal failure: Consensus definitions and overview. Clin. Gastroenterol. Hepatol..

[3] Pironi L., Arends J., Bozzetti F., Cuerda C., Gillanders L., Jeppesen P.B., Joly F., Kelly D., Lal S., Staun M. (2016). ESPEN guidelines on chronic intestinal failure in adults. Clin. Nutr..

[4] Pironi L., Cuerda C., Jeppesen P.B., Joly F., Jonkers C., Krznarić Ž., Lal S., Lamprecht G., Lichota M., Mundi M.S. (2023). ESPEN guideline on chronic intestinal failure in adults—Update 2023. Clin. Nutr..

[5] Nightingale J., Woodward J.M., Small Bowel and Nutrition Committee of the British Society of Gastroenterology (2006). Guidelines for management of patients with a short bowel. Gut.

[6] Iyer K., DiBaise J.K., Rubio-Tapia A. (2022). AGA Clinical Practice Update on Management of Short Bowel Syndrome: Expert Review. Clin. Gastroenterol. Hepatol..

[7] Baethge C., Goldbeck-Wood S., Mertens S. (2019). SANRA—A scale for the quality assessment of narrative review articles. Res. Integr. Peer Rev..

[8] Woolf G.M., Miller C., Kurian R., Jeejeebhoy K.N. (1983). Diet for patients with a short bowel: High fat or high carbohydrate?. Gastroenterology.

[9] McIntyre P.B., Fitchew M., Lennard-Jones J.E. (1986). Patients with a high jejunostomy do not need a special diet. Gastroenterology.

[10] Jeppesen P.B., Mortensen P.B. (1998). The influence of a preserved colon on the absorption of medium chain fat in patients with small bowel resection. Gut.

[11] Newton C.R., Gonvers J.J., McIntyre P.B., Preston D.M., Lennard-Jones J.E. (1985). Effect of different drinks on fluid and electrolyte losses from a jejunostomy. J. R Soc. Med..

[12] Nightingale J.M., Lennard-Jones J.E., Walker E.R., Farthing M.J. (1992). Oral salt supplements to compensate for jejunostomy losses: Comparison of sodium chloride capsules, glucose electrolyte solution, and glucose polymer electrolyte solution. Gut.

[13] Jeppesen P.B., Staun M., Tjellesen L., Mortensen P.B. (1998). Effect of intravenous ranitidine and omeprazole on intestinal absorption of water, sodium, and macronutrients in patients with intestinal resection. Gut.

[14] Jeppesen P.B., Gilroy R., Pertkiewicz M., Allard J.P., Messing B., O’Keefe S.J. (2011). Randomised placebo-controlled trial of teduglutide in reducing parenteral nutrition and/or intravenous fluid requirements in patients with short bowel syndrome. Gut.

[15] Jeppesen P.B., Pertkiewicz M., Messing B., Iyer K., Seidner D.L., O’Keefe S.J., Forbes A., Heinze H., Joelsson B. (2012). Teduglutide reduces need for parenteral support among patients with short bowel syndrome with intestinal failure. Gastroenterology.

[16] Jeppesen P.B., Vanuytsel T., Subramanian S., Joly F., Wanten G., Lamprecht G., Kunecki M., Rahman F., Nielsen T.S., Berner-Hansen M. (2025). Glepaglutide, a long-acting glucagon-like peptide-2 analogue, reduces parenteral support in patients with short bowel syndrome: A phase 3 randomized controlled trial. Gastroenterology.

[17] Eliasson J., Hvistendahl M.K., Freund N., Bolognani F., Meyer C., Jeppesen P.B. (2022). Apraglutide, a novel glucagon-like peptide-2 analog, improves fluid absorption in patients with short bowel syndrome intestinal failure: Findings from a placebo-controlled, randomized phase 2 trial. JPEN J. Parenter. Enter. Nutr..

[18] Joly F., Vanuytsel T., Kirby D.F., Lal S., Tappenden K., Jeppesen P.B., Youssef N.N., Li C., Sheik R., Statovci I. (2024). Efficacy and safety of apraglutide once-weekly in patients with short bowel syndrome and intestinal failure (SBS-IF): Results from the STARS study, a global phase 3 double-blind, randomized, placebo-controlled trial. Clin. Nutr. ESPEN.

[19] Pironi L., Boeykens K., Bozzetti F., Joly F., Klek S., Lal S., Lichota M., Mühlebach S., Van Gossum A., Wanten G. (2020). ESPEN guideline on home parenteral nutrition. Clin. Nutr..

[20] Pironi L., Boeykens K., Bozzetti F., Joly F., Klek S., Lal S., Lichota M., Mühlebach S., Van Gossum A., Wanten G. (2023). ESPEN practical guideline: Home parenteral nutrition. Clin. Nutr..

[21] Alfonso J.E., Berlana D., Ukleja A., Boullata J. (2017). Clinical, ergonomic, and economic outcomes with multichamber bags compared with (hospital) pharmacy compounded bags and multibottle systems: A systematic literature review. JPEN J. Parenter. Enter. Nutr..

[22] Jones D., Allsopp K., Sowerbutts A.M., Lal S., Farrer K., Harrison S., Burden S. (2024). Outcomes for patients receiving multi-chamber bags for the delivery of parenteral nutrition: A systematic review. Nutrients.

[23] Mundi M.S., Martindale R.G., Calder P.C., Klek S. (2025). Long-term/home parenteral nutrition: Expert consensus statements regarding intravenous lipid emulsions. Front. Nutr..

[24] Cavicchi M., Beau P., Crenn P., Degott C., Messing B. (2000). Prevalence of liver disease and contributing factors in patients receiving home parenteral nutrition for permanent intestinal failure. Ann. Intern. Med..

[25] Vahedi K., Atlan P., Joly F., Le Brun A., Evard D., Perennec V., Roux-Haguenau D., Bereziat G., Messing B. (2005). A 3-month double-blind randomised study comparing an olive oil- with a soyabean oil-based intravenous lipid emulsion in home parenteral nutrition patients. Br. J. Nutr..

[26] Klek S., Chambrier C., Singer P., Rubin M., Bowling T., Staun M., Joly F., Rasmussen H., Strauss B.J., Wanten G. (2013). Four-week parenteral nutrition using a third generation lipid emulsion (SMOFlipid): A double-blind, randomised, multicentre study in adults. Clin. Nutr..

[27] Klek S., Chambrier C., Szczepanek K., Kunecki M., Sobocki J., Wanten G., Pironi L., Schneider S.M., Rahman F., Cooper S.C. (2024). Safe and well-tolerated long-term parenteral nutrition regimen: Omega-3-fatty-acid-enriched medium chained/long chained triglycerides emulsion. Clin. Nutr..

[28] Matarese L.E., Jeppesen P.B., O’Keefe S.J.D. (2014). Short bowel syndrome in adults: The need for an interdisciplinary approach and coordinated care. JPEN J. Parenter. Enter. Nutr..

[29] Buchman A.L., Scolapio J., Fryer J. (2003). AGA technical review on short bowel syndrome and intestinal transplantation. Gastroenterology.

[30] Nightingale J.M., Lennard-Jones J.E., Walker E.R., Farthing M.J. (1990). Jejunal efflux in short bowel syndrome. Lancet.

[31] Nightingale J.M., Lennard-Jones J.E., Gertner D.J., Wood S.R., Bartram C.I. (1992). Colonic preservation reduces need for parenteral therapy, increases incidence of renal stones, but does not change high prevalence of gall stones in patients with a short bowel. Gut.

[32] Nordgaard I., Hansen B.S., Mortensen P.B. (1994). Colon as a digestive organ in patients with short bowel. Lancet.

[33] Nordgaard I., Hansen B.S., Mortensen P.B. (1996). Importance of colonic support for energy absorption as small-bowel failure proceeds. Am. J. Clin. Nutr..

[34] Tappenden K.A. (2014). Pathophysiology of short bowel syndrome: Considerations of resected and residual anatomy. JPEN J. Parenter. Enter. Nutr..

[35] Carbonnel F., Cosnes J., Chevret S., Beaugerie L., Ngô Y., Malafosse M., Parc R., Le Quintrec Y., Gendre J.P. (1996). The role of anatomic factors in nutritional autonomy after extensive small bowel resection. JPEN J. Parenter. Enter. Nutr..

[36] Prahm A.P., Brandt C.F., Askov-Hansen C., Mortensen P.B., Jeppesen P.B. (2017). The use of metabolic balance studies in the objective discrimination between intestinal insufficiency and intestinal failure. Am. J. Clin. Nutr..

[37] Crenn P., Morin M.C., Joly F., Penven S., Thuillier F., Messing B. (2004). Net digestive absorption and adaptive hyperphagia in adult short bowel patients. Gut.

[38] Matarese L.E., O’Keefe S.J., Kandil H.M., Bond G., Costa G., Abu-Elmagd K. (2005). Short bowel syndrome: Clinical guidelines for nutrition management. Nutr. Clin. Pract..

[39] Jeppesen P.B., Fuglsang K.A. (2018). Nutritional Therapy in Adult Short Bowel Syndrome Patients with Chronic Intestinal Failure. Gastroenterol. Clin. N. Am..

[40] Crenn P., Coudray-Lucas C., Thuillier F., Cynober L., Messing B. (2000). Postabsorptive plasma citrulline concentration is a marker of absorptive enterocyte mass and intestinal failure in humans. Gastroenterology.

[41] Pogatschnik C., Russell L. (2025). Dietary interventions for short bowel syndrome in adults. Nutrients.

[42] Mayeur C., Gratadoux J.J., Bridonneau C., Chegdani F., Larroque B., Kapel N., Corcos O., Thomas M., Joly F. (2013). Faecal D/L lactate ratio is a metabolic signature of microbiota imbalance in patients with short bowel syndrome. PLoS ONE.

[43] Pironi L., Morselli Labate A.M., Pertkiewicz M., Przedlacki J., Tjellesen L., Staun M., De Francesco A., Gallenca P., Guglielmi F.W., Van Gossum A. (2002). Prevalence of bone disease in patients on home parenteral nutrition. Clin. Nutr..

[44] Jeppesen P.B., Sanguinetti E.L., Buchman A., Howard L., Scolapio J.S., Ziegler T.R., Gregory J., A Tappenden K., Holst J., Mortensen P.B. (2005). Teduglutide (ALX-0600), a dipeptidyl peptidase IV resistant glucagon-like peptide 2 analogue, improves intestinal function in short bowel syndrome patients. Gut.

[45] Schwartz L.K., O’Keefe S.J., Fujioka K., Gabe S.M., Lamprecht G., Pape U.F., Li B., Youssef N.N., Jeppesen P.B. (2016). Long-term teduglutide for the treatment of patients with intestinal failure associated with short bowel syndrome. Clin. Transl. Gastroenterol..

[46] Jeppesen P.B., Gabe S.M., Seidner D.L., Lee H.M., Olivier C. (2018). Factors associated with response to teduglutide in patients with short-bowel syndrome and intestinal failure. Gastroenterology.

[47] Naimi R.M., Hvistendahl M., Enevoldsen L.H., Madsen J.L., Fuglsang S., Poulsen S.S., Kissow H., Pedersen J., Nerup N., Ambrus R. (2019). Glepaglutide, a novel long-acting glucagon-like peptide-2 analogue, for patients with short bowel syndrome: A randomised phase 2 trial. Lancet Gastroenterol. Hepatol..

[48] Iyer K., Joly F., Kirby D., Lal S., Tappenden K., Jeppesen P., Youssef N.N., Boules M. (2024). Phase 3, double-blind, randomized STARS trial of apraglutide once-weekly in patients with short bowel syndrome and intestinal failure (SBS-IF): Subgroup analyses by baseline demographics and SBS disease characteristics [abstract S2211]. Am. J. Gastroenterol..

[49] Jeppesen P., Tappenden K., Kirby D., Lal S., Vanuytsel T., Ming C., Masior T., Boules M., Joly F., Iyer K. (2025). Long-term treatment with once-weekly apraglutide reduces parenteral support dependency and allows to reach enteral autonomy in some patients with short bowel syndrome and intestinal failure: The STARS Extend trial [abstract S3026]. Am. J. Gastroenterol..

[50] Jeppesen P.B., Pertkiewicz M., Forbes A., Pironi L., Gabe S., Joly F., Messing B., Loth S., Youssef N., Heinze H. (2013). Quality of life in patients with short bowel syndrome treated with the new glucagon-like peptide-2 analogue teduglutide: Analyses from a randomised, placebo-controlled study. Clin. Nutr..

[51] Zafirovska M., Zafirovski A., Rotovnik Kozjek N. (2023). Current insights regarding intestinal failure–associated liver disease (IFALD): A narrative review. Nutrients.

[52] Lal S., Pironi L., Wanten G., Arends J., Bozzetti F., Cuerda C., Joly F., Kelly D., Staun M., Szczepanek K. (2018). Clinical approach to the management of intestinal failure associated liver disease (IFALD) in adults: A position paper from the Home Artificial Nutrition and Chronic Intestinal Failure Special Interest Group of ESPEN. Clin. Nutr..

[53] Covello C., Becherucci G., Di Vincenzo F., Del Gaudio A., Pizzoferrato M., Cammarota G., Gasbarrini A., Scaldaferri F., Mentella M.C. (2024). Parenteral nutrition, inflammatory bowel disease, and gut barrier: An intricate plot. Nutrients.

[54] Picot D., Layec S., Seynhaeve E., Dussaulx L., Trivin F., Carsin-Mahe M. (2020). Chyme reinfusion in intestinal failure related to temporary double enterostomies and enteroatmospheric fistulas. Nutrients.

[55] Messing B., Crenn P., Beau P., Boutron-Ruault M.C., Rambaud J.C., Matuchansky C. (1999). Long-term survival and parenteral nutrition dependence in adult patients with the short bowel syndrome. Gastroenterology.

[56] Iyer K.R. (2014). Surgical management of short bowel syndrome. JPEN J. Parenter. Enter. Nutr..

[57] Grant D., Abu-Elmagd K., Mazariegos G., Vianna R., Langnas A., Mangus R., Farmer D.G., Lacaille F., Iyer K., Fishbein T. (2015). Intestinal transplant registry report: Global activity and trends. Am. J. Transplant..

[58] Wouters Y., Theilla M., Singer P., Tribler S., Jeppesen P.B., Pironi L., Vinter-Jensen L., Rasmussen H.H., Rahman F., Wanten G.J.A. (2018). Randomised clinical trial: 2% taurolidine versus 0.9% saline locking in patients on home parenteral nutrition. Aliment Pharmacol. Ther..

[59] Cuerda C., Pironi L., Arends J., Bozzetti F., Gillanders L., Jeppesen P.B., Joly F., Kelly D., Lal S., Staun M. (2021). ESPEN practical guideline: Clinical nutrition in chronic intestinal failure. Clin. Nutr..

[60] Joly F., Baxter J., Staun M., Kelly D.G., Hwa Y.L., Corcos O., De Francesco A., Agostini F., Klek S., Santarpia L. (2018). Five-year survival and causes of death in patients on home parenteral nutrition for severe chronic and benign intestinal failure. Clin. Nutr..

[61] Amiot A., Messing B., Corcos O., Panis Y., Joly F. (2013). Determinants of home parenteral nutrition dependence and survival of 268 patients with non-malignant short bowel syndrome. Clin. Nutr..

[62] Joly F., Mayeur C., Bruneau A., Noordine M.-L., Meylheuc T., Langella P., Messing B., Duée P.-H., Cherbuy C., Thomas M. (2010). Drastic changes in fecal and mucosa-associated microbiota in adult patients with short bowel syndrome. Biochimie.

[63] Budinska E., Gojda J., Heczkova M., Bratova M., Dankova H., Wohl P., Bastova H., Lanska V., Kostovcik M., Dastych M. (2020). Microbiome and metabolome profiles associated with different types of short bowel syndrome: Implications for treatment. JPEN J. Parenter. Enter. Nutr..

[64] Gillard L., Mayeur C., Robert V., Pingenot I., Le Beyec J., Bado A., Lepage P., Thomas M., Joly F. (2017). Microbiota is involved in post-resection adaptation in humans with short bowel syndrome. Front. Physiol..

